# Modified Dynamic Conformal Arcs With Forward Planning for Radiosurgery of Small Brain Metastasis: Each Double Arc and Different To-and-Fro Leaf Margins to Optimize Dose Gradient Inside and Outside the Gross Tumor Boundary

**DOI:** 10.7759/cureus.34831

**Published:** 2023-02-10

**Authors:** Kazuhiro Ohtakara, Kojiro Suzuki

**Affiliations:** 1 Department of Radiation Oncology, Kainan Hospital Aichi Prefectural Welfare Federation of Agricultural Cooperatives, Yatomi, JPN; 2 Department of Radiology, Aichi Medical University, Nagakute, JPN

**Keywords:** stereotactic radiosurgery, small tumor, micro-multileaf collimator, forward planning, dynamic conformal arcs, dose inhomogeneity, dose gradient, dose distribution, brain metastasis, brain invasion

## Abstract

Dynamic conformal arcs (DCA) are a widely used technique for stereotactic radiosurgery (SRS) of brain metastases (BM) using a micro-multileaf collimator (mMLC), while the planning design and method considerably vary among institutions. In the usual forward planning of DCA, the steepness of the dose gradient outside and inside the gross tumor volume (GTV) boundary is simply defined by the leaf margin (LM) setting to the target volume edge. The dose fall-off outside the small GTV tends to be excessively precipitous, especially with an MLC of 2.5-mm leaf width, which is predisposed to the insufficient coverage of microscopic brain invasion and other inherent inaccuracies. Meanwhile, insufficient dose increase inside the GTV boundary, i.e., less inhomogeneous GTV dose, likely leads to inferior and less sustainable tumor response. The more inhomogeneous GTV dose is prone to the steeper dose gradient outside the GTV and vice versa. Herein, we describe an alternative simply modified DCA (mDCA) planning that was uniquely devised to optimize the dose gradient outside and inside the GTV boundary for further enhancing and consolidating local control of small BM.

For a succinct exemplification, a 10-mm spherical target was assumed as a GTV for DCA planning using a 2.5-mm mMLC. The benchmark plan was generated by adding a 0-mm LM to the GTV edge by assigning a single fraction of 30 Gy to the isocenter, in which the GTV coverage by 24 Gy with 80% isodose surface (IDS) was 96%, i.e., D_96%_, while the coverage of GTV + isotropic 2 mm volume by 18 Gy with 60% IDS was 70%, with the D_98%_ being 12 Gy with 40% IDS, viz., too steep dose fall-off outside the GTV boundary. Alternatively, the increase of LM with or without decreasing the isocenter dose enables the increase of the GTV + 2 mm coverage by 18 Gy while resulting in an inadequate GTV dose with either a less inhomogeneous dose or an excessive marginal dose. Meanwhile, in the newly devised mDCA planning, every single arc was converted to a double to-and-fro arc with different LM settings under the same spatial arrangement, which enabled GTV + 2 mm volume coverage with 18 Gy while preserving the GTV marginal dose and inhomogeneity similar to those for the benchmark plan. Additionally, the different collimator angle (CA) setting for the to-and-fro arcs led to further trimming of the dose conformity.

The limitations of general forward planning with only adjusting the LM for every single arc were demonstrated, which can be a contributing factor for local tumor progression of small BM. Alternatively, the mDCA with each double to-and-fro arc and different LM and CA settings enables optimization of the dose gradient both outside and inside the GTV boundary according to the planners’ intent, e.g., moderate dose spillage margin outside the GTV and steep dose increase inside the GTV boundary.

## Introduction

Stereotactic radiosurgery (SRS) is an indispensable treatment option as focal therapy for brain metastases (BM), concertedly with anti-cancer pharmacotherapy [1,2]. Long-term local control (LC) and safety have been unprecedentedly anticipated for a substantial number of BM cases, especially for cases with isolated central nervous system failure without the extracranial active disease who are not scheduled to receive any anti-cancer medication that can enhance anti-BM efficacy and/or soothe adverse radiation effect [3,4]. LC failure and/or symptomatic irreversible radiation injury following SRS of just a single BM can inflict a devastating effect on the patient’s health and life. Local definitive and radical treatment far beyond palliation is demanded by a certain proportion of BM patients, for whom scrupulous and conscientious SRS design and planning are needed [5].

In a substantial number of BM, the brain-BM interfaces are pathologically poorly demarcated, viz., the presence of microscopic brain infiltration with various depths and patterns as a function of the BM volume and histopathology [6]. Complete tumor eradication, including brain invasion, is demanded to achieve superior long-term LC. Consequently, a steep dose gradient outside the gross tumor volume (GTV) boundary can lead to insufficient dose coverage for brain invasion [5,7]. Therefore, moderate, not too precipitous or gradual, dose attenuation outside the GTV would be desirable, also given the other treatment-related uncertainties, including intra-fractional patient movement [5]. Thus, the ideal and optimal dose distribution of SRS for BM would be considerably different from those for pathologically well-demarcated benign tumors and vascular malformation. Meanwhile, a steep dose increase inside the GTV boundary, i.e., an extremely inhomogeneous GTV dose can also be beneficial to enhance anti-BM efficacy by conquering the potential internal radioresistant portion and is also deemed as another essential integrant for suitable dose distribution [5,7]. Several studies suggest the advantage of so-called internal dose escalation for effecting superior tumor shrinkage and LC [8,9]. Thus, in addition to the prescribed marginal dose, the appropriateness of the dose gradient both outside and inside the GTV boundary is an indispensable requisite for SRS planning for BM [5].

Dynamic conformal arcs (DCA) is a commonly used forward planning technique for linac-based SRS using a micro-multileaf collimator (mMLC) [3,10]. DCA is dosimetrically robust and straightforward to validate for planning and delivery. Meanwhile, there have been substantial inter-institutional differences and variability regarding target definition, prescription dose, and target dose inhomogeneity [7]. The optimal planning design and method remain unestablished. SRS of small-sized BM of <1 cm^3^ is particularly expected to achieve a high probability of long-term LC and safety, given the limited tumor infiltration and lower susceptibility to brain injury [2,4,11]. However, >10% of BM of <1 cm^3^ present local tumor progression (LTP) after SRS within two years [2,4,11]. In DCA for small BM, especially with the smaller leaf width mMLC, such as 2.5 mm, the dose gradient outside the GTV margin tends to be extremely steep [7,12]. Meanwhile, expanding the dose spillage margin outside the GTV likely leads to impaired GTV dose and its inhomogeneity with either a lower inhomogeneous dose or excessive GTV marginal dose.

In this report, we describe the limitations of general forward planning of DCA, with only adjusting leaf margin (LM) to the target volume edge for every single arc in terms of optimizing the dose distribution, along with the potential detrimental implications on anti-BM efficacy. We then demonstrate a simple and convenient alternative forward planning method as a solution, which was devised to arbitrarily optimize the degree of the dose gradient both outside and inside the GTV boundary, according to the planners’ intent and strategy.

The synopsis of this report was previously presented at the 33^rd^ Annual Meeting of the Japanese Society for Radiation Oncology held online on October 1-3, 2020.

## Technical report

In this study, frameless SRS in a single fraction with DCA for small-sized BM was assumed, for which the intended dose distribution included as follows: 1) GTV was covered by 24 Gy with ≤80% isodose surface (IDS), normalized to 100% at the isocenter of ≥30 Gy, and the GTV coverage of ≥98%, i.e., D_≥98%_; 2) the boundary of the GTV + isotropic 2 mm volume was covered by 18 Gy with ≤60% IDS and the coverage of 95-98%, D_95-98%_, to fully compensate for inherent treatment-related uncertainties [4,5,13-15]. Meanwhile, planning target volume (PTV) is usually generated by adding a 1-mm margin to GTV [4,7,12]. A single fraction of 24 Gy provides approximately 95% of one-year local tumor control probability in which the GTV coverage of ≥98% is a common standard [13,14]. The treatment platform used in this study was Novalis Truebeam STx® (Palo Alto, CA: Varian Medical Systems) with a flattening filter (FF) free mode of a 6 megavoltage (MV) X-ray beam, equipped with an integrated high-definition mMLC, i.e., HD120, which has a central leaf width of 2.5 mm [12,16]. The dosimetric characteristics of usual DCA with HD120 and an FF mode and the differences in those between 2.5-mm and its predecessor 3-mm leaf width were described previously [10,12]. The treatment planning system was iPlan Dose (Munich, Germany: Brainlab AG) with a calculation grid size of 1 mm in the then-version, for which forward planning was only available for DCA [7,12].

Benchmark plan

**Figure 1:**
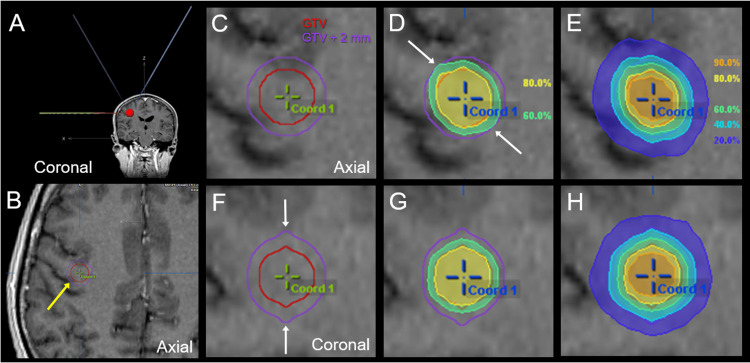
Benchmark plan: Arc arrangement, target definition, and dose distributions. The images show an arc arrangement (A); target definition (B, C, F); an enlarged view of B (C); dose distributions (D, E, G, H); coronal views (A, F-H); and axial views (B-E). (A) The arc arrangement consists of one coplanar arc and two non-coplanar arcs with each arc length of 140º and collimator angle of 0º, unless otherwise specified, to divide the cranial hemisphere evenly. (C, F) The gross tumor volume (GTV) was defined as a sphere of 10 mm in diameter that was automatically generated with the treatment planning system, which is susceptible to the voxel size of computerized tomographic images with a 1.25-mm slice thickness. The GTV + 2 mm volume for evaluation is generated by adding an isotropic 2-mm margin to the GTV. In the coronal view, both the GTV and GTV + 2 mm volume shapes are awkwardly irregular (arrows in F). (D, E, G, H) Represented % isodoses are normalized to 100% at the isocenter of 30 Gy. (D, E) The dose spillage near the GTV boundary, e.g., 60 and 80% isodose lines, in the axial views is unevenly and disproportionately distributed (arrows in D) compared to those for the coronal view (G, H).

The arc arrangement, target definition with a model spherical target, and dose distributions of the benchmark plan for DCA are shown in Figure 1.

A 10-mm spherical target was assumed as the GTV. In the benchmark plan, the LM to the GTV edge was set to isotropic 0 mm, in which 30 Gy was assigned to the isocenter. Consequently, the GTV coverage with 24 Gy (80% IDS) was 96%, while the GTV + 2 mm volume coverage with 18 Gy (60% IDS) was 70%, with the D_98%_ being 12 Gy (40% isodose), that is, too steep dose fall-off outside the GTV boundary (Figures 1D, 1G).

Alternative plan A

**Figure 2:**
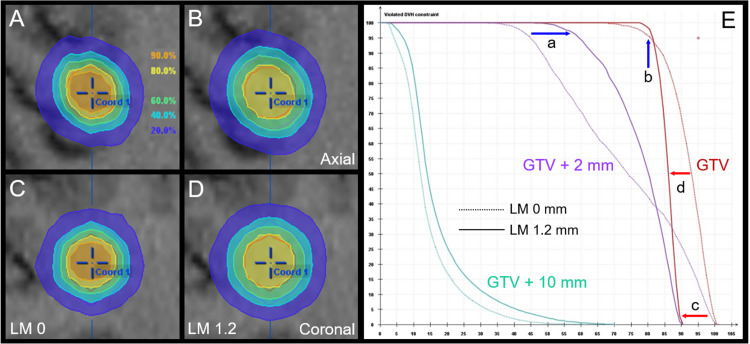
Alternative plan A: Dose distributions and dose-volume histograms compared to those for the benchmark plan. The images show dose distributions (A-D); dose-volume histograms (DVH) (E); axial views (A, B); coronal views (C, D); benchmark plan (A, C); and alternative plan A (B, D). (A-D) Represented isodoses are normalized to 100% at the isocenter dose of 30 Gy for the benchmark plan, in which 26.67 Gy is assigned to the isocenter dose for alternative plan A. (E) The GTV + 2 mm volume coverage with 18 Gy isodose increased to 97% (a), and the GTV coverage with 24 Gy increased to 98% (b) in alternative plan A. However, the maximum (c) and median (d) doses of the GTV decreased in alternative plan A. The GTV + 10 mm volume represents total irradiated isodose volumes, including GTV. LM X: Leaf margin X mm; GTV: Gross tumor volume

To increase the GTV + 2 mm volume coverage with 18 Gy isodose, the LM to the GTV edge was expanded to isotropic 1.2 mm, and the isocenter dose was reduced to 26.67 Gy, which resulted in the GTV + 2 mm volume coverage with 18 Gy (67.4% IDS) increasing to 97%, and the GTV coverage with 24 Gy (90% IDS) increasing to 98% compared to the benchmark plan. However, the GTV D_50%_ decreased by 7%, which was contrary to internal dose escalation (Figure 2).

Alternative plan B

**Figure 3:**
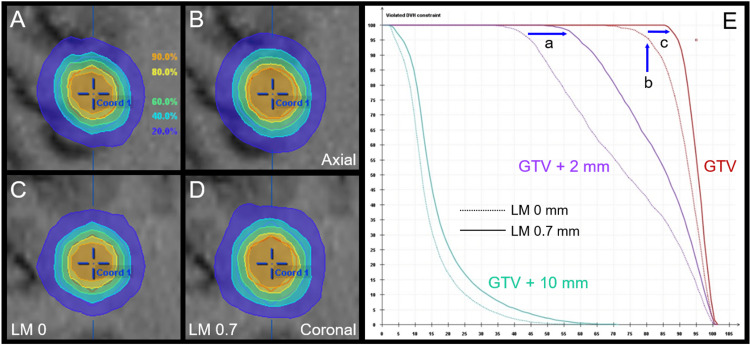
Alternative plan B: Dose distributions and dose-volume histograms compared to those for the benchmark plan. The images show dose distributions (A-D); DVH (E); axial views (A, B); coronal views (C, D); benchmark plan (A, C); and alternative plan B (B, D). (A-D) Represented isodoses are normalized to 100% at the isocenter dose of 30 Gy for the benchmark plan and alternative plan B. (E) The GTV + 2 mm volume coverage with 18 Gy increased to 95% (a), and the GTV coverage with 24 Gy become too excessive, (b) with the GTV D_98%_ increasing to 26.3 Gy in alternative plan B. Meanwhile, the maximum dose is preserved. LM X: Leaf margin X mm; GTV: Gross tumor volume; DVH: Dose-volume histograms; D_98%_: A minimum dose encompassing at least 98% of the object volume

To increase the GTV + 2 mm coverage with 18 Gy isodose (60% IDS) while preserving the isocenter dose, the LM to the GTV edge was set to 0.7 mm, which resulted in the GTV + 2 mm coverage with 18 Gy increasing to 95%. However, 24 Gy isodose with 80% IDS to the GTV edge showed over-coverage with the GTV D_98%_ increasing to 26.3 Gy, which renders the surrounding brain susceptible to higher dose exposure while enhancing anti-tumor effects (Figure 3).

Modified DCA with each double arc and different to-and-fro leaf margins

**Figure 4:**
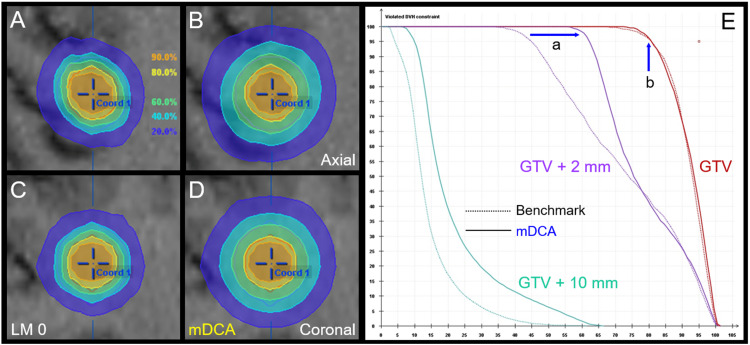
Modified dynamic conformal arcs: Dose distributions and dose-volume histograms compared to those for the benchmark plan. The images show dose distributions (A-D); DVH (E); axial views (A, B); coronal views (C, D); benchmark plan (A, C); and modified dynamic conformal arcs (mDCA) (B, D). (A-D) Represented isodoses are normalized to 100% at the isocenter dose of 30 Gy for the benchmark plan and mDCA. (E) The GTV + 2 mm volume coverage with 18 Gy increased to 98% (a), and the GTV coverage with 24 Gy (b) and the median and maximum doses are preserved in the mDCA. (A, B) The disproportionate distributions of 60% and 80% IDS are improved in the mDCA. LM 0: Leaf margin 0 mm; GTV: Gross tumor volume; DVH: Dose-volume histograms

To improve the intrinsic detriments for alternative plans A and B, every single arc was changed to double to-and-fro arcs, and the collimator angles for each arc were differently set to 45º and 315º for to- and fro- arcs, respectively, while the isocenter dose was preserved to 30 Gy. The LM was also differently set to -0.5 mm and 3.0 mm for to- and fro- arcs, respectively. The beam weights, i.e., monitor unit (MU) for each arc, were evenly allocated basically, which can be unevenly allocated if necessary. Consequently, the GTV + 2 mm volume coverage with 18 Gy (60% IDS) increased to 98%, while the GTV coverage with 24 Gy (80% IDS) along with the internal doses was preserved. Additionally, the skewed distributions of 60%-80% IDS relative to the GTV edge for the benchmark plan were also improved to be more concentrically laminated (Figure 4).

**Figure 5:**
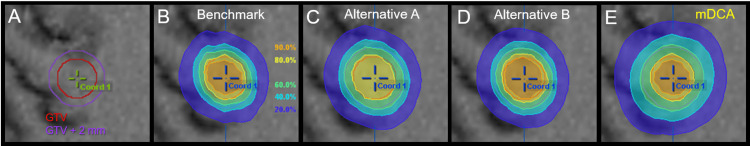
Comparative view of four differently optimized plans for dynamic conformal arcs. The images show defined target volumes (A); dose distributions (B-E); benchmark plan (B); alternative plan A (C); alternative plan B (D); and modified dynamic conformal arcs (mDCA) (E). (B-E) Representative % isodoses are normalized to 100% at the isocenter of 30 Gy for the benchmark plan. GTV: Gross tumor volume

For convenient and objective comparison, the axial dose distributions of the four presented plans are shown in Figure 5, and the dosimetric parameters are tabulated in Table 1.

**Table 1:** Dosimetric parameters of four differently optimized plans for dynamic conformal arcs. LM: Leaf margin; GTV: Gross tumor volume; % IDS: % Isodose surface normalized to 100% at the isocenter; D98%: A dose encompassing at least 98% of the object volume; mDCA: Modified dynamic conformal arcs

Plan	LM to GTV	Isocenter dose	% IDS of 24 Gy	GTV coverage with 24 Gy	GTV D_98%_	% IDS of 18 Gy	PTV coverage with 18 Gy
Benchmark	0 mm	30 Gy	80%	96%	23.0 Gy	60%	70%
Alternative A	1.2 mm	26.67 Gy	90%	98%	24.0 Gy	67.4%	97%
Alternative B	0.7 mm	30 Gy	80%	100%	26.3 Gy	60%	95%
mDCA	-0.5 / 3.0 mm	30 Gy	80%	96%	23.4 Gy	60%	98%

## Discussion

Leksell Gamma Knife (LGK) has been one of the mainstays for implementing SRS of BM since its first clinical application in 1975 [17]. The LTP rate following single-fraction SRS with LGK for BM of <1 cm^3^ exceeded >10% within two years in the JLGK0901 study, in which the LTP was defined as a ≥20% increase in the maximum diameter of the enhanced lesion compared to that for the maximum response for BM of >1 cm [11]. The prescribed marginal dose of <22 Gy was one of the significant contributing factors for LTP [11]. The pathological condition of a ≥20% increase of an enhanced lesion following the nadir response can include regrowth of the residual tumor and radiation injury of the surrounding brain, i.e., adverse radiation effect (ARE) [3,5]. The possible causes responsible for incomplete tumor necrosis include the following: 1) insufficient GTV marginal dose; 2) insufficient GTV internal dose increase; 3) too steep of a dose fall-off outside the GTV boundary; and 4) inherent setup inaccuracy even for frame-based immobilization [5,16,18]. Regarding the irradiation accuracy, cone-beam computerized tomography (CBCT)-based verification recently disclosed that the mean value of the initial residual setup error was 0.8 ± 0.4 mm in 3D vector length, with the maximum being 2.0 mm, which has been perennially overlooked until the emergence of integrated CBCT [18]. Although generally unregarded dose attenuation margin outside the prescription IDS can cover these uncertainties to some degree, an approximately 1-mm setup error likely leads to marginal tumor residues, given the generally steep dose gradient for LGK [5]. Meanwhile, recent studies also suggest that the higher proportion of GTV receiving ≥30-32 Gy in the single fraction, i.e., internal dose escalation, is likely associated with superior tumor shrinkage and LC [8,9,19]. In LGK, 50% IDS is generally used for target coverage and dose prescription in multi-shot planning, i.e., a combination of multi-isocenter, which can lead to superior tumor response, particularly if concentrically laminated steep dose increase inside the GTV boundary is achieved [5,11]. However, single-shot treatment is rather common for small-sized BM, especially for simultaneous treatment of multiple BM. For single-shot planning for various-sized BM, one size of the collimator is frequently chosen to cover each GTV, among the extremely limited size of collimators available for LGK, usually 8 mm or 14/16 mm according to the version. Consequently, even when the marginal dose is equivalent, the dose inhomogeneities of each GTV, along with the maximum dose substantially, vary as a function of the GTV size and/or collimator size, in which some BM can be covered by >80-90% IDS, i.e., rather a homogeneous GTV dose similar to those for some linac-based SRS. When the GTV boundary is covered by 22 Gy with 80-90% IDS, the maximum dose ranges from 24.4 to 27.5 Gy, viz. <30 Gy, which is contrary to the preferred internal dose escalation, and GTV D_98%_ is prone to be <24 Gy [11,13,14]. In the same-day irradiation of multiple BM with LGK, the difference and variability of GTV dose inhomogeneity can lead to different tumor responses. However, in almost all of the previous reports regarding LGK for multiple BM, the SRS plans have been described only as a so-called marginal dose with specific % IDS covering a single representative GTV, in which the GTV dose inhomogeneities for other GTVs remained substantially varied and inherently uncertain [2,11,13-15]. Thus, we consider indispensable prerequisites for SRS planning to achieve ≥95% of ≥ 2-year sustained tumor regression for small BM as follows: 1) GTV marginal dose of ≥24 Gy; 2) concentrically laminated steep dose increase inside the GTV boundary; and 3) moderate dose spillage margin outside the GTV [5,13,14].

Compared to the aforementioned issues and limitations for LGK, mMLC-based DCA enables generation of a variably sized and shaped conformal irradiation field in 0.1-mm increment to various target volumes [10,12]. Therefore, DCA planning enables equalization of dose inhomogeneities for each target volume, e.g., 80% IDS coverage, more uniformly compared to those for LGK [7]. Meanwhile, the degree of the dose gradient outside the GTV is similarly susceptible to the % IDS adopted for target coverage and dose interference if multiple targets are simultaneously irradiated [7]. In usual forward planning of DCA, the steepness of the dose gradient outside the GTV boundary is requisitely specified according to the LM setting to the target volume edge [7,12]. The dose fall-off outside the small GTV tends to be excessively precipitous, especially with an MLC of 2.5-mm leaf width [12]. Meanwhile, insufficient dose increase inside the GTV boundary, i.e., less inhomogeneous GTV dose, likely leads to inferior and less sustainable tumor response [15,19]. The more inhomogeneous GTV dose is prone to the steeper dose gradient outside the GTV and vice versa [7].

Recently, dedicated software, namely Elements Multiple Brain Mets SRS (Munich, Germany: Brainlab AG), has provided inverse planning-based DCA optimization with a single isocenter for either single or multiple BM [20]. However, in this inverse planning, the dose distribution is optimized by designating a prescription dose to a specific % IDS covering either GTV or margin-added PTV, along with the coverage value. Compared to volumetric-modulated arc therapy, the latitude of adjusting the dose gradient both outside and inside the GTV boundary is considerably restricted. Particularly, in simultaneous irradiation of multiple targets through a single isocenter setting, a target volume located more distant from the isocenter is likely more susceptible to intra-fractional rotational error. Thus, caution should be exercised to avoid excessively precipitous dose fall-off outside the GTV. Meanwhile, an excessively large setup margin inevitably leads to an increased risk of ARE [3,4]. Therefore, moderate, not too steep or gradual, dose spillage or attenuation margin outside the GTV should be considered.

We should embrace attention to the limitations of general forward planning with only adjusting LM for every single arc and the potential detrimental implications on LC [7,10,12]. The newly devised mDCA enables optimization of the dose gradient both outside and inside the GTV boundary, i.e., moderate dose spillage margin outside the GTV and steep dose increase inside the GTV boundary. Different dose distributions presented in this report (Figure 5) inevitably exert different effects on both the BM and surrounding normal tissue. Which is the most suitable for long-term LC and safety depends on pathological conditions such as radiosensitivity and/or invasiveness. In terms of minimizing the dose to the normal tissue, the benchmark plan would be beneficial, while alternative plan B with a higher GTV dose may be preferable for radioresistant histology. Given the aforementioned benefit of internal dose escalation, GTV coverage with ≤70% IDS may be further advantageous [5,7]. The different to-and-fro LM settings can be adjusted with various combinations along with the beam weights, according to the planner’s intent and preferences.

The presented results warrant further investigation to verify the dosimetric advantages for various clinical scenarios and also determine whether the presented mDCA reduces LTP without increasing ARE for small-sized BM.

## Conclusions

In DCA for SRS of small-sized BM, the limitations of general forward planning with only adjusting LM for every single arc and the potential detrimental implications on LC should be considered. Alternatively, the mDCA with each double to-and-fro arc and different LM and CA settings enables optimization of the dose gradient both outside and inside the GTV boundary, i.e., moderate dose spillage margin outside the GTV and steep dose increase inside the GTV boundary. Furthermore, the presented mDCA enables arbitrary optimization of the dose distribution according to the planners’ intent to be tailored to suit each clinical condition.
